# Predictors of mortality in HIV-associated hospitalizations in Portugal: a hierarchical survival model

**DOI:** 10.1186/1472-6963-9-125

**Published:** 2009-07-23

**Authors:** Sara S Dias, Valeska Andreozzi, Maria O Martins, Jorge Torgal

**Affiliations:** 1 Higher Institute of Statistics and Information Management, New University of Lisbon, Lisbon, Portugal; 2 Center of Statistics and Applications of Lisbon University, Lisbon, Portugal; 3 Public Health University Department, Medical Sciences Faculty, New University of Lisbon, Lisbon, Portugal; 4 Institute of Hygiene and Tropical Medicine, New University of Lisbon, Lisbon, Portugal

## Abstract

**Background:**

The beneficial effects of highly active antiretroviral therapy, increasing survival and the prevention of AIDS defining illness development are well established. However, the annual Portuguese hospital mortality is still higher than expected. It is crucial to understand the hospitalization behaviour to better allocate resources. This study investigates the predictors of mortality in HIV associated hospitalizations in Portugal through a hierarchical survival model.

**Methods:**

The study population consists of 12,078 adult discharges from patients with HIV infection diagnosis attended at Portuguese hospitals from 2005–2007 that were registered on the diagnosis-related groups' database.

We used discharge and hospital level variables to develop a hierarchical model. The discharge level variables were: age, gender, type of admission, type of diagnoses-related group, related HIV complication, the region of the patient's residence, the number of diagnoses and procedures, the Euclidean distance from hospital to the centroid of the patient's ward, and if patient lived in the hospital's catchment area. The hospital characteristics include size and hospital classification according to the National Health System. Kaplan-Meier plots were used to examine differences in survival curves. Cox proportional hazard models with frailty were applied to identify independent predictors of hospital mortality and to calculate hazard ratios (HR).

**Results:**

The Cox proportional model with frailty showed that male gender, older patient, great number of diagnoses and pneumonia increased the hazard of HIV related hospital mortality. On the other hand tuberculosis was associated with a reduced risk of death. Central hospital discharge also presents less risk of mortality.

The frailty variance was small but statistically significant, indicating hazard ratio heterogeneity among hospitals that varied between 0.67 and 1.34, and resulted in two hospitals with HR different from the average risk.

**Conclusion:**

The frailty model suggests that there are unmeasured factors affecting mortality in HIV associated hospitalizations. Consequently, for healthcare policy purposes, hospitals should not all be treated in an equal manner.

## Background

The introduction of highly active antiretroviral therapy (HAART) in late 1996 dramatically improved the prognosis of Human Immunodeficiency Virus (HIV) infected patients in most developed countries [1,2]. Consequently, the estimated number of worldwide deaths due to HIV/AIDS is declining, which is attributable to the scaling up of antiretroviral treatment services. Nevertheless, HIV/AIDS remains a leading cause of mortality throughout the world. In Portugal, although the access to treatment has been free of charge since 1996 and available in all hospitals since 2005, there are still an elevated number of deaths due to HIV/AIDS, namely, in men between 25 and 34 years old [3]. Despite declining mortality over the last years, adult HIV prevalence in 2007 ranges from 0.1% to 0.6% in Europe. Portugal is one of the European countries with the highest prevalence (0.5%) [4], where 31,667 cases of HIV/AIDS were notified in 2007 [5].

HIV/AIDS is one of the major financial burdens on healthcare systems worldwide. In the United States the average annual HIV/AIDS charge per patient was estimated to be approximately $18,000 from 1996–2000 [6]. In Portugal, hospitalizations related to HIV infection are also some of the most expensive; the average daily cost is around €825 and it is the second major diagnosis category (MDC) with greater average hospitalization time. The average length of stay (LOS) in Portuguese hospitals from the National Health Service (NHS) was 23 days in 2006 [7,8]. Although this value is very high compared to countries like the USA or Canada, where the average is around 5 days [6,9], it is similar to European countries such as Germany where the average LOS is 20 days [10].

In recent literature several approaches have been adopted to analyse LOS. Barbour et al. [6] used a multivariable linear regression model to study charges among HIV/AIDS inpatients. On the other hand, Huang et al. [11] used a generalized linear mixed model to study LOS and costs, and Wang et al. [12] adopted a two-component Poisson mixture model to analyse maternity LOS. In the present study, LOS will be considered the main vehicle that allows the study of hospital mortality of HIV infected patients.

In the study of hospital mortality one has to be aware that the risk of death cannot be assumed to be homogeneous, but must be considered as a heterogeneous, i.e. a mixture of individuals with different hazards. In this particular case, the hospital mortality may vary according to individual patient and hospital characteristics. The former can be taken into account by including covariates at the hospitalization level in a classical Cox proportional survival model. Differences in the health services can be assessed using hierarchical models, including variables at hospitalization and hospital levels, allowing the estimate of differences in outcome not fully explained by observed patient or other specific and known conditions. The aim of this paper is to investigate predictors of mortality in HIV associated hospitalizations in Portugal. We propose the use of a hierarchical time survival model to analyse data of HIV discharges in Portuguese hospitals from 2005 to 2007.

To our knowledge, this is the first study using this type of methodology for HIV discharges in Portugal. This analysis is particularly important given the considerable burden associated with HIV treatment/hospitalization.

## Methods

### Data source

The data were provided by the Central Administration of Health System (ACSS) and refer to the Portuguese national database of the diagnosis related groups (DRG – All Patients v21.0). The data are anonymous and available from the ACSS for scientific research. In the DRG database each record corresponds to a discharge episode (hospitalization) and contains information about the patient and information collected while the patient was in hospital.

Hospital characteristics were obtained from data provided by ACSS and the Portuguese National Institute of Statistics.

### Study Population

Between 1^st ^January 2005 and 31^st ^December 2007 there were 14,165 discharges registered in Portuguese NHS hospitals. For this study we considered only those that met the following criteria: hospitalizations classified under the MDC 24 created for HIV infections patients, which incorporates the DRG 700–716; inpatients aged 18 years or older; geo-referenced cases, i.e., hospitalization with patient residence ward known; hospitalizations from hospitals with more than 10 discharges; all hospitalizations were included except those for transfers to another hospital (to avoid including the inpatient episode twice, given that often the cause of the transfer was lack of procedure facilities); and hospitalizations with LOS ≥ 1 day.

**Figure 1:**
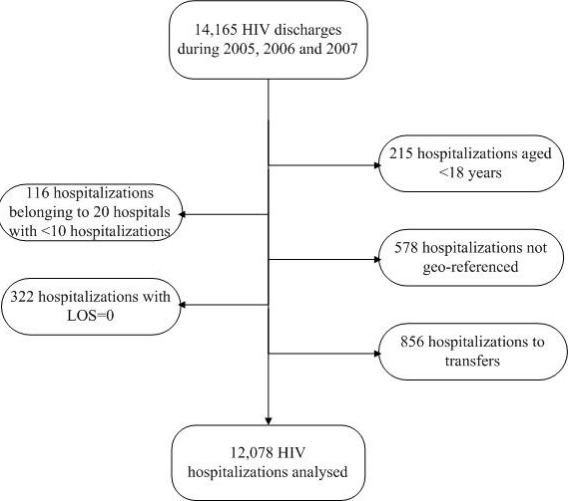
**Selection profile of study population**.

With these criteria, we selected 12,078 hospitalizations occurring in 43 public NHS hospitals (Figure 1).

### Dependent variable

The dependent variable was the number of days between the hospital admission and discharge dates (LOS). Death was assumed to be the outcome of interest and censoring was considered when patients were alive at discharge.

### Covariates

#### Discharge level variables

The discharge variables considered in this study were: the age of the patient on admission, gender, the type of admission (urgent or planned), the type of DRG (medical or surgical), the HIV infection related complication (pneumonia, tuberculosis, pneumocystis pneumonia (PCP), hepatitis B and C), the patient's residential region (North, Centre, Lisbon and Tagus valley, and South). Specific data on income, education, and other components of socioeconomic status (SES) were not available. Therefore, we utilized the type of admission variable as a proxy for SES as in Barbour et al. [6]. The Euclidean distance from hospital to the centroid of the patient's residential ward in kilometres was calculated. Whether the patient lived in the catchment area of the hospital was another distance covariate. In addition, the number of diagnoses and the number of procedures undertaken were also available for each hospitalization. In the exploratory analysis, age, number of diagnoses and procedures were divided into two categories according to their median value (age: ≤ 39, >39 years; number of diagnoses ≤ 5, >5; and number of procedures ≤ 8, >8). The distance was categorized according to its average value of 13 km.

#### Group-level variables (hospital characteristics)

The following information was available to characterize the NHS hospital: the hospital size represented by the number of beds (500 beds maximum and greater than 500 beds); classifications according to NHS administrative structure (hospitals offering more differentiated services denoted central).

### Statistical analysis

Descriptive statistics were computed for all variables: means, medians, and standard deviations for continuous variables, and frequencies and percentages for categorical variables.

Kaplan-Meier (KM) estimators were applied to estimate survival curves, and log rank tests and Peto tests were used for comparison between variables categories.

Cox proportional hazard models [13] were used to identify independent predictors of hospital mortality and calculate hazard ratios (HR).

To take into account the left truncation of the survival discharge data we adopted the Cox model with a counting process approach and also included random effects to deal with covariates hierarchy denoted frailty models. This random effect can be thought of as a "frailty", increasing hospital's susceptibility to short survival time when it is large, and decreasing this susceptibility when it is small.

The equation λ (t|**x**)= *zλ*_0_(t)exp(**xβ**) describes the frailty model where **x **are the covariates matrix, β are the fixed effect vector, and Z is a random variable representing an unknown random effect, and related to hospitals, with unit mean and variance ξ. These random effects act multiplicatively on the baseline hazard, and large values of ξ reflect a great degree of heterogeneity among hospitals. Groups with frailty >1 tend to experience the event at a faster rate than under the basic Cox model, indicating in our case a poor hospital performance. On the other hand, when frailty is <1, survival times tend to be longer. It should be noted that when the variance of *Z *approaches zero, the model reduces to the basic Cox model. For model distribution purposes, we assumed that the frailties were distributed according to a gamma distribution [14]. One attractive feature of the gamma distribution is that it is mathematically tractable. Frailty models were treated as a penalized Cox model [15] and were estimated using the survival package from R software.

To build the model, we first included in a classic Cox survival model only the hospitalization level and group level variables which were statistically significant at the 10% level in KM analysis; thereafter the random effects were added. To compare the Cox model and the Frailty model, we applied the likelihood ratio (LR) test. All the statistical analyses were performed using R statistical software [16].

## Results

In this study the median LOS was equal to 12 days (interquartile range 6–24). Summary statistics of the hospitalization according to discharge episodes and hospital characteristics are in Table 1.

**Table 1:** Characteristics of HIV discharges in Portuguese NHS hospitals

**Variables **(mean ± sd)	**n = 12,078**
Age; years	41.6 ± 11.5
LOS; days	19.2 ± 24.8
Number of diagnoses	6.2 ± 3.5
Number of procedures	8.2 ± 4.3
Gender (%)	
Male	8,954 (74.1)
Female	3,124 (25.9)
Year of discharge (%)	
2005	4,111 (34.0)
2006	4,124 (34.2)
2007	3,843 (31.8)
Type of admission (%)	
Urgent	10,281 (85.1)
Planned	1,797 (14.9)
Type of DRG (%)	
Medical	11,192 (92.7)
Surgical	886 (7.3)
Pneumonia (%)	2,401 (19.9)
Tuberculosis (%)	2,256 (18.8)
PCP (%)	892 (7.4)
Hepatitis B (%)	483 (4.0)
Hepatitis C (%)	2,896 (24)

Hospital size (beds)	538.3 ± 360.1
Hospital catchment area (%)	
Live in	8,998 (74.5)
Live out	3,080 (25.5)
Hospital classification (%)	
Central	8,206 (68.0)
Non-central	3,872 (32.0)

Out of 12,078 discharges, 8,954 (74.1%) were male and the median age was 42 years. The number of discharges decreased 7.8% in the last year of the study. There were 10,281 (85.1%) urgent admissions and the majority of the discharges (92.7%) had medical DRG. Over the three years of study, the most common HIV related complications were pneumonia and tuberculosis, accounting for 19.9% and 18.7% of the discharges, respectively. The endpoint death occurred in 15.2% (1,833) of the hospitalizations.

**Figure 2:**
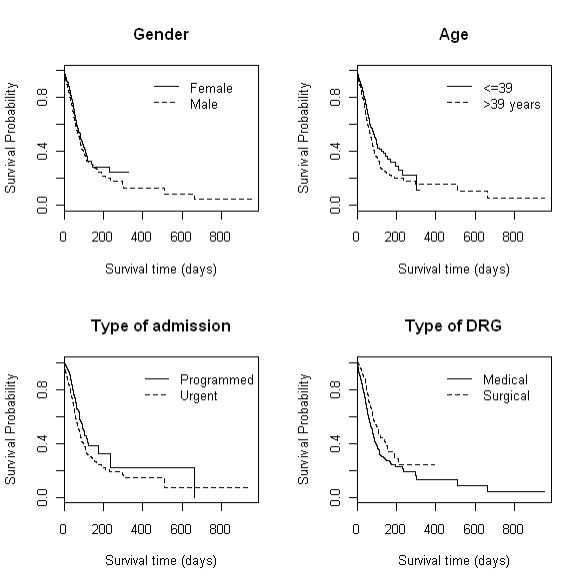
**KM estimates of survival for selected variables: gender, age, type of admission and type of DRG**.

KM exploratory analysis suggests that hospitalization from older patients, men, urgently admitted, and those with medical DRG were associated with a faster progression to death (Figure 2).

For these four variables log rank and Peto tests for KM survival differences were all highly significant (p < 0.001). Hospitalization with a greater number of diagnoses and procedures also presented lower survival. The main HIV related infection complication risk factor for survival was pneumonia, which is also statistically significant, as can be seen from Table 2. Patients with tuberculosis showed a high median survival LOS and this was also statistically significant. There was no statistically significant difference between presences or absence of PCP and hepatitis B. Hepatitis C was statistically significant at the 10% level. The year of discharge and the patient's residential region did not yield significant survival differences. Both Euclidean distance and catchment area presented statistically significant differences in KM survival curves. However, because they are highly associated and the former did not represent the real distance between hospital and patient residence, living in the hospital catchment area was chosen to be included in the survival model as a proxy of residential distance to hospital.

**Table 2:** Kaplan-Meier survival estimator of 12,078 HIV hospitalizations

**Variables**	**Median survival LOS (95% CI)**	**p-value Log Rank test**	**p-value Log Rank test**
Gender			
Male	75 (69; 80)	<0.001	<0.001
Female	84 (73; 104)		
Year of discharge			
2005	76 (71; 87)	0.357	0.405
2006	78 (69; 100)		
2007	75 (68; 88)		
Age			
≤ 39	92 (83; 104)	<0.001	<0.001
> 39	71 (65; 76)		
Type of admission			
Urgent	75 (69; 80)	<0.001	<0.001
Planned	95 (77;-)		
Type of DRG			
Medical	75 (69; 80)	<0.001	<0.001
Surgical	110 (84; 191)		
Number of diagnoses			
≤ 5	83 (74;-)	0.040	0.080
> 5	75 (69; 80)		
Number of procedures			
≤ 8	100 (80; 140)	0.175	0.175
> 8	75 (69; 80)		
Pneumonia			
Yes	53 (48; 59)	<0.001	<0.001
No	87 (81; 100)		
Tuberculosis			
Yes	97 (83; 113)	<0.001	<0.001
No	69 (64; 78)		
PCP			
Yes	87 (78;140)	0.162	0.179
No	76 (71; 83)		
Hepatitis B			
Yes	80 (50;-)	0.412	0.275
No	77 (73; 83)		
Hepatitis C			
Yes	80 (75;100)	0.092	0.112
No	76 (70; 83)		
Region			
North	75 (64; 85)	0.258	0.193
Centre	78 (68; 135)		
Lisbon and Tagus valley	78 (70; 84)		
South	- (74;-)		
Distance			
≤ 13 km	75 (69; 80)	0.008	0.023
> 13 km	92 (75; 170)		
Hospital size			
N. beds ≤ 500	76 (70; 84)	0.089	0.078
N. beds > 500	79 (72; 85)		
Hospital catchment area			
Live in	75 (69;79)	0.009	0.014
Live out	87 (78; 110)		
Hospital classification			
Central	82 (75; 88)	<0.001	<0.001
Non-central	71 (61; 79)		

The two hospital characteristics provided KM survival curves differences statistically significant at the 10% level.

The variables thus selected and included in the model were: gender, age, type of admission, type of DRG, number of diagnoses, number of procedures, pneumonia, tuberculosis, hepatitis C, living in hospital catchment area, hospital size, and hospital classification. Table 3 shows the HR for each covariate in the Cox survival models with and without frailty. From the frailty model, men increase the risk, as expected, by 27.0% in comparison to women. Each additional year of age leads to an increase in risk of 1.3%. Urgent admission presents the highest risk, increasing it by 60% in comparison with programmed admission. Surgical DRG reduces the risk of death by 44%. Each additional diagnosis raises the hospital mortality risk by only 2%. Pneumonia is the most serious HIV related infection complication, raising the risk by 43%, while tuberculosis presents a protective effect, reducing the risk by 22%. Hepatitis C and live in hospital catchment area were not important predictors to hospital mortality. Parameter estimates of individual covariates are quite similar for both models.

**Table 3:** Cox proportional hazards models for hospital mortality

	**No frailty**		**Frailty**	
**Variables**	**HR (95% CI)**	**p-value**	**HR (95% CI)**	**p-value**
Gender (male)	1.268 (1.13; 1.42)	<0.001	1.272 (1.14; 1.43)	<0.001
Age (years)	1.013 (1.01; 1.02)	<0.001	1.013 (1.01; 1.02)	<0.001
Type of admission (urgent)	1.699 (1.43; 2.02)	<0.001	1.592 (1.33; 1.91)	<0.001
Type of DRG (surgical)	0.569 (0.47; 0.69)	<0.001	0.560 (0.46; 0.68)	<0.001
Number of diagnoses	1.018 (1.00; 1.03)	0.012	1.020 (1.01; 1.04)	0.012
Number of procedures	0.997 (0.98; 1.01)	0.580	0.994 (0.98;1.01)	0.360
Pneumonia	1.428 (1.29; 1.59)	<0.001	1.431 (1.29; 1.59)	<0.001
Tuberculosis	0.779 (0.69; 0.88)	<0.001	0.783 (0.69; 0.88)	<0.001
Hepatitis C	1.013 (0.90; 1.14)	0.820	1.011 (0.90; 1.14)	0.850
Hospital size (>500 beds)	1.118 (1.01; 1.24)	0.033	1.145 (0.93; 1.41)	0.210
Live in catchment area	1.032 (0.92; 1.15)	0.590	1.011 (0.90; 1.14)	0.850
Central hospital	0.798 (0.72; 0.89)	<0.001	0.808 (0.68; 0.97)	0.002

Frailty variance	0.032			0.002

Regarding hospital covariates, the hospital size presents statistical significance only in the Cox model without frailty. Nonetheless, large hospitals (>500 beds) present a hazard of 1.14 greater than smaller hospitals. The central hospital presents statistically significant difference from non-central hospital in both models and can be considered to be a protective factor, decreasing the hazard by 20%.

**Figure 3:**
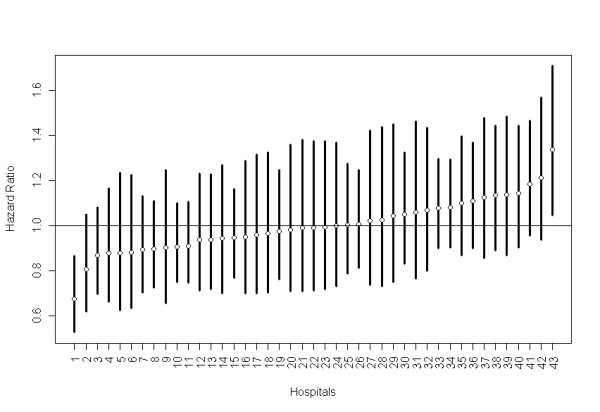
**Frailty model estimates of HR of hospitals showing the point estimate (circle) and their respective 95% CI band**.

The inclusion of a frailty effect expands the coefficient 95% confidence intervals of the hospital characteristics, and the hospital size loses statistical significance. The estimated frailty variance (0.032) is statistically significant (p = 0.002) and indicates the presence of a small variability among Portuguese NHS hospitals (Figure 3). The hospitals specific HRs, varying from 0.67 to 1.34, are presented in Figure 3 with their respective 95% confidence interval. Notably, there is only one hospital with frailty significantly greater than 1 and there is also one hospital with frailty significantly less than 1. Hospital number 1 (with frailty significantly less than 1) corresponds to a large teaching central hospital in a metropolitan area, while hospital number 43 (with frailty significantly greater than 1) is a non-central hospital in a smaller area.

## Discussion

To our knowledge, this is the first paper that applies hierarchical modelling to account for hospital and individual characteristics among HIV/AIDS discharges in Portugal. Risk factors for mortality in the frailty model included male gender, older age, urgent admission, medical DRG, a higher number of diagnoses, pneumonia, and admission to a non-central hospital.

The discharge variables included in the model behaved as expected. In Portuguese NHS hospitals, as well as in those of other countries [17,18], men are associated with a higher risk of death due to HIV/AIDS than are women. Urgent admission presents the highest hazard ratio. Considered as a proxy for SES, this finding confirms the results obtained in previous studies, where patients with urgent admission are of lower SES when compared to patients with programmed admission [19,20]. On the contrary, we find that surgical hospitalizations yield the lowest risk rates, reducing the risk of death by 44%. This result was somehow expected since the patients hospitalized to surgical procedures usually have a well established clinical situation, better than a patient with a non-surgical hospitalization. In consonance with, the double percentage of planned admission in surgical hospitalization compared with the one in non-surgical hospitalization can be used to strengthen this hypothesis. We also have to consider the fact that DRG data is designed to be a financial system which determines how hospital will be reimbursed. The predetermined DRG reimbursement is based on principal diagnosis, procedures, patient age and sex, the presence of complications or comorbidities, surgical intervention and, discharge type. However, this homogeneous form of calculating the hospital reimbursement causes serious financial losses to hospital when the primary mode of therapy associated with the hospitalization is surgical. A surgical procedure will imply the use of an operating room which will have a great influence on the hospital means used by the patient and in the final costs [21]. Accordingly, the DRG type was introduced in Cox model to control the effect of DRG case mix.

Pneumonia was also a strong predictor of hospital mortality in our study. Patients with pneumonia were 1.43 times more likely to die when compared to those without pneumonia. This finding is in accordance with studies from Europe [22] and North America [23] showing that pneumonia is an important predictor of mortality in patients with HIV/AIDS.

On the other hand, tuberculosis has been associated with improved survival [24-27]. In our study, tuberculosis was also associated with better hospital survival, reducing the risk of death by 22%. This protective effect relies on the fact that tuberculosis incidence rate (34.4/100,000 inhabitants) in Portugal is one of the highest in Europe [28] and, it is the first disease diagnosed in HIV infection patients. Therefore, tuberculosis HIV patients are hospitalized sooner than other patients and begin HAART treatment earlier.

The issue of whether hepatitis C effects progression of HIV-disease remains controversial [29]. In the Swiss Cohort, the presence of hepatitis C was independently associated with an increased risk of progression to AIDS and death [30]. However subsequent studies from other cohorts, did not find any differences in survival when multivariate analysis was applied. For example, the EuroSIDA cohort found no difference between hepatitis C positive and hepatitis C negative HIV-infected patients [31]. In our study, hepatitis C is not associated with hospital mortality after adjustment for other covariates.

The number of diagnoses and procedures for a particular patient express the complications and co-morbidities that the patient had during hospital stay. A great number of diagnoses or procedures usually indicates a more severe condition of the patient [32]. In this analysis only the number of diagnoses was statistically significant increasing the mortality risk by 2% for each increase in the number of diagnoses.

Hospital size was measured in terms of number of beds. Lee and collaborators [33] have shown that efficient hospitals had fewer beds than inefficient hospitals, everything else being constant. Based on this evidence, we expected that smaller hospital had a lower risk of death. However, in our study, hospital size only suggests a statistically significant increase in hospital mortality risk in the Cox Model without frailties.

The present study found that there are significant differences in hospital mortality due to hospital classification. The risk of death is reduced by 20% in a central hospital when compared to a non-central hospital. This finding is somewhat expected since in Portugal the follow-up of patients with HIV/AIDS is more frequent in central hospitals, where anonymity is guaranteed; often, patients only attend non-central health services when they are in a serious acute situation. Additionally, it is well known that dedicated AIDS units and management hospitals offer important benefits to AIDS patients [34] and in Portugal, central hospitals are the only ones that have communicable diseases department.

Living in the catchment hospital area was used as a proxy of residential distance to hospital based on the evidence that increased distance was associated with increased risk of death [35-37]. However, in our study this variable was not statistically significant in both models.

The frailty model estimated a small variability among hospitals (range 0.67 to 1.34), although it is statistically significant. Standard single-level models, usually adopted in these studies, treat all hospitalizations as independent observations. Actually, patients are nested in hospitals on the basis of reasons that lead them to make some choices (place of residence, trust in a particular doctor, the hospital's reputation, etc), thus violating one of the basic assumptions of traditional regression analysis. In hierarchical models the random variability of data is divided into two parts: variation between different hospitalizations and between different hospitals. The hospital frailty component should be interpreted as differences in hospital quality/performance.

Consequently, hierarchical modelling is strongly advocated as a more appropriate statistical method for dealing with data clustered within hospitals [38-42]. This is the one of the strengths of our study, taking into account the hierarchical nature of the DRG data.

The frailty model revealed a variation in risk not resulting from any of the measured covariates. One possible explanation for this variability might be the differences in hospital accessibility and services availability. Due to the inexistence of a variable for measuring the accessibility, one way to incorporate this could be through the investigation of a geographical pattern in the frailty of hospitals [42,43]. In this study, we used the catchment area, but this variable was not statistically significant in the frailty model. Additional research is needed here.

### Study limitations

This study, like most studies, has limitations. The DRG data cannot account for multiple admissions, reducing our experimental unit to discharge episode instead of patient. It should be stressed that the information on hospital characteristics was gathered for other purposes than this study and some vital features of HIV/AIDS patients are missing, such as CD4 counts and viral load [9,44].

## Conclusion

The use of DRG data is crucial in comparing different hospitals, patients, and places. Moreover, the frailty model used in this study proved to be useful for this purpose. Nonetheless, it is a strong candidate to be a very efficient tool for the state health authorities, due to software availability. Accounting for clustering of observations, the frailty model provides truthful estimates of standard errors and assures less biased results. Our findings suggested that, for healthcare policy purposes, the hospitals should not be treated in an equal manner; namely the one identified as outlier.

For future developments we intend to construct a joint spatial survival model to investigate factors that affect HIV hospital mortality and taking into account geographical factors. This work can contribute to the debate on the evaluation of a hospital's performance on HIV treatment. Also, our results might help hospital managers/practitioners understand the major predictors of HIV related mortality and to look at it in a different way.

Finally, local investigation of higher risk hospitals should be undertaken in order to disclose factors leading to differences in the HIV mortality risk detected in this study.

## Competing interests

The authors declare that they have no competing interests.

## Authors' contributions

SSD, VA and MOM performed the statistical analysis and helped to draft the manuscript. JT helped to draft the manuscript. All authors read and approved the final manuscript.

## Pre-publication history

The pre-publication history for this paper can be accessed here:


